# *FAM153A* Is a Novel Biomarker of Human Thermogenic Adipocytes

**DOI:** 10.3390/cells15151321

**Published:** 2026-07-24

**Authors:** Katalin Gyurina, Kristóf Levente Korpás, Anita Bajusz-Rácz, Ádám Radványi, László Sasi-Szabó, Gábor Méhes, Tamás Röszer

**Affiliations:** 1 Department of Pediatrics, Faculty of Medicine, University of Debrecen, Nagyerdei krt. 98, 4032 Debrecen, Hungary; 2 Department of Pathology, Faculty of Medicine, University of Debrecen, Nagyerdei krt. 98, 4032 Debrecen, Hungary; 3 Institute of Materials and Environmental Chemistry, Research Centre for Natural Sciences, 1117 Budapest, Hungary

**Keywords:** obesity, thermogenesis, beige adipocyte, brown adipocyte, adipose tissue

## Abstract

**Highlights:**

- *FAM153A* is a novel mRNA marker of thermogenic fat.
- qPCR-based analysis of *FAM153A* may indicate thermogenic competence.
- *FAM153A* outperforms *UCP1* as a marker of thermogenic transcriptional identity.

**Abstract:**

Childhood obesity may be associated with an accelerated loss of thermogenic adipocytes, increasing the risk of progressive lipid accumulation and metabolic deterioration. Early detection of the loss of thermogenic adipocytes would allow improved intervention to abrogate childhood obesity, yet reliable markers of human thermogenic adipocytes remain limited. We previously identified expression of *FAM153A* (family with sequence similarity 153 member A) in the developing human adipose tissue. Here, we investigated the expression pattern and potential association of *FAM153A* with the thermogenic transcriptional program in the adipose tissue of infants, children, and adolescents. We found that adipocyte *FAM153A* expression was triggered by beta adrenergic receptor stimulation, a key signal of adipocyte thermogenesis. *FAM153A* protein was confined to preadipocyte and adipocyte nuclei, and *FAM153A* mRNA expression positively correlated with the mRNA levels of canonical thermogenic adipocyte markers including uncoupling protein 1 (*UCP1*), myogenic differentiation 1 (*MYOD1*), type II iodothyronine deiodinase (*DIO2*), transmembrane protein 26 (*TMEM26*), Lim Homeobox 8 (*LHX8*), beta adrenergic receptor 2 (*ADRB2*), and peroxisome proliferator-activated receptor gamma coactivator 1-alpha (*PPARGC1A*). Importantly, *FAM153A* expression was positively associated not only with the expression of individual thermogenic genes but also with their coordinated co-expression. Collectively, our findings identify *FAM153A* as a novel mRNA biomarker of human thermogenic adipocytes that serves as a robust indicator of the thermogenic transcriptional network.

## 1. Introduction

Living with obesity as a child increases the risk of developing chronic diseases, such as metabolic syndrome, type 2 diabetes mellitus, cardiovascular diseases, hepatic steatosis, autoimmunity and chronic inflammatory conditions, and certain types of malignancies in early adulthood [1,2]. Early diagnosis is crucial to prevent the development of these diseases since early intervention may protect the patient from further metabolic deterioration [3]. Moreover, pediatric obesity has a heterogenous etiology, and children with similar anthropometric values—such as body weight, body mass index (BMI), or weight-for- age percentiles—may have substantially different metabolic risks, treatment responses, and underlying pathophysiology [4,5,6,7]. The effectiveness of weight management programs hence largely depends on individual risk assessment, precise identification of the underlying causes of obesity, and personalized treatment plans [4,5,6,7]. There is a demand for precision medicine in pediatric obesity diagnosis and treatment; however, adipose tissue development and functioning are still largely unexplored in children [8,9].

Precision medicine depends heavily on molecular biology analysis of biopsies, which provides information on the metabolic activity of the adipose tissue and deciphers gene networks and mechanisms that might be responsible for the development of obesity or may respond to a certain intervention [9]. For instance, compromised thermogenic potential is an important contributor to the development of obesity [10], especially in children [11,12,13]. Thermogenic adipocytes—often termed as beige, brown, or brite adipocytes—appear in the subcutaneous adipose tissue in the third trimester of the intrauterine life and persist throughout childhood, with increasing activity as puberty advances [12,14,15]. They are important players in maintaining core body temperature after birth by utilizing fatty acids to produce heat in uncoupled mitochondrial respiration [16]. Later in life they help to maintain healthy glucose control and limit the availability of lipogenic substrates in adipocytes as they utilize carbohydrate and fatty acid fuel [12]. The amount of thermogenic fat is proportional to the skeletal muscle mass in adolescents [11,17,18,19], and the loss of thermogenic fat is an early sign of obesity development in children [12,15,20]. Premature loss of thermogenic fat in infancy may lead to obesity in childhood by increasing lipid storage because of diminished thermogenesis [15,21]. Estimating thermogenic competence of fat depots is hence important in the context of assessing obesity risk in childhood. Uncoupling protein 1 (UCP1) is considered today as a hallmark of thermogenic adipocytes [22], since it allows mitochondrial uncoupling and thermogenesis. However, UCP1-independent thermogenesis may also occur [23], and thermogenic adipocyte identity may be better defined by measuring the expression of a set of genes necessary to thermogenic adipocyte functioning [24]. It is key, therefore, to establish quantitative, measurable, sensitive and specific indicators, so-called biomarkers, that reflect the thermogenic adipocyte content of the adipose tissue without the need to measure the expression of a set of genes.

Elective surgical procedures provide access to adipose tissue specimens that are routinely discarded as surgical waste but can be repurposed for histological, transcriptomic, proteomic, and biomarker analyses, thereby enabling direct assessment of adipose tissue biology and function [25,26]. We have recently described the expression of *FAM153A*, encoding family with sequence similarity 153, member A in the developing human adipose tissue [15]. It is also known as renal carcinoma antigen NY-REN-7. In this study, we continued to explore its expression pattern in the adipose tissue of infants, children, and adolescents. We show that *FAM153A* mRNA serves as an indicator of thermogenic adipocytes. It is more robustly expressed than *UCP1* mRNA and is closely related to the thermogenic adipocyte transcriptional identity.

## 2. Materials and Methods

### 2.1. Human Adipose Tissue Samples

Subcutaneous adipose tissue samples were obtained from the abdominal-inguinal region of infants, children, and adolescents undergoing elective surgical procedures at the Department of Pediatrics, University of Debrecen. Biopsies were analyzed from 125 patients (112 males, 13 females) ranging in age from 2 months to 17.4 years. Anthropometric and clinical parameters, including body weight, height, and medical history, were systematically recorded and evaluated. Fat specimens were used for RNA isolation, histology, and flow cytometry analysis. Inclusion and exclusion criteria were applied according to the protocol previously described [15]. Written informed consent was obtained from the parents or legal guardians of all participants prior to sample collection. The study was conducted in accordance with the ethical principles outlined in the Declaration of Helsinki. We considered sex as a biological variable, and we recruited patients from both sexes. However, due to sex-dependent differences in the clinical indications of surgical interventions, male patients were overrepresented in our cohorts. Fetal specimens (between intrauterine 17th and 38th weeks) were collected during autopsies performed at the Department of Pathology, University of Debrecen, and samples were used for histology analysis.

### 2.2. Treatment of Human Adipocytes

We isolated primary human adipocytes from biopsies collected during elective surgeries of patients between 6 months and 4 years of age, using collagenase digestion [27], and cells were maintained in high-glucose Dulbecco’s modified Eagle medium (DMEM) supplemented with 10% fetal bovine serum, 1% L-glutamine, and 20 µg/mL of insulin (I9278, Merck, St. Louis, MO, USA). Adipocytes were treated with 100 μM isoproterenol (15627, Merck) for 4 h.

### 2.3. RNA Isolation and qPCR

We used TRIzol reagent (T9424-100 mL, Merck, St. Louis, MO, USA) for extraction of total RNA, according to the manufacturer’s protocol. We used NanoDrop 2000/2000c spectrophotometer (1.6.198, Thermo Fisher Scientific, Waltham, MA, USA) to assess RNA concentration and purity. A high-capacity cDNA reverse transcription kit was used to synthesize cDNA (4368813, Thermo Fisher Scientific). qPCR assays were conducted using the Analytik Jena platform. Relative gene expression levels were calculated using the mean threshold cycle (Ct) values of the reference genes *ACTB* and *GAPDH* and are presented on a logarithmic scale. Primer sequences are shown in Table S1. Specification of the qPCR primer pair used for detecting *FAM153A* is shown in Tables S2 and S3. Amplification efficiency for each primer was determined from the log-linear phase of individual amplification curves using the LinRegPCR method [28].

### 2.4. Histology, Immunohistochemistry, and Flow Cytometry

We fixed adipose tissue specimens in 4% paraformaldehyde (PFA) prepared in phosphate-buffered saline (PBS). Specimens were embedded in paraffin, then sectioned and stained with hematoxylin and eosin. Adipocyte area was measured with ImageJ (2.16.0/1.54p Java 1.8.0_452, NIH, USA). Prior to histomorphometry analysis, image calibration was performed analyzing a known reference distance based on the embedded scale bar within each JPG image. To normalize intensity values across samples, we converted images to 8-bit grayscale format. Threshold adjustment was applied to optimize contrast and facilitate accurate identification of cellular structures of interest. Individual cells were manually outlined.

We used a rabbit polyclonal antibody against *FAM153A* protein (HPA044022, 1:50 dilution, Merck) to detect *FAM153A* in formalin-fixed, paraffin-embedded tissue sections, using citric acid-based antigen unmasking solution for antigen retrieval (H-3300-250, Vector Laboratories, Newark, CA, USA). Antibody binding was visualized using an HRP-conjugated secondary antibody followed by chromogenic detection with Impact DAB (Vector Laboratories).

For flow cytometry analysis, we used adipose tissue biopsies collected from patients between 6 months and 4 years of age. Adipocytes and the stromal vascular fraction cells were isolated by collagenase digestion of the biopsies. Adipocytes were fixed in 0.5% PFA, permeabilized with eBioscience permeabilization buffer (Thermo Fisher Scientific), labeled with the same *FAM153A* antibody used for immunohistochemistry, and labeled with a fluorescent secondary antibody (F-2765, Thermo Fisher Scientific). Lipid droplets were labeled with HCS LipidTOX™ Red Neutral Lipid Stain (H34476, Thermo Fisher Scientific). Adipogenic cells were labeled with an antibody against cluster of differentiation 36 (CD36) [29] (MA1-10210, Thermo Fisher Scientific), while adipocytes were labeled with an antibody against perilipin (PLIN1) [30] (690156S, Thermo Fisher Scientific), in combination with an AF488 conjugated secondary antibody, also from Thermo Fisher Scientific. Flow cytometric analyses were performed using a BD LSR II Flow Cytometer (BD Biosciences, Franklin Lakes, NJ, USA), with FACS Diva v8.0 (BD Biosciences) software for analysis (Figure S1).

### 2.5. Statistical Analyses

Gene expression values were transformed as log_10_(x + ε), where ε = 1 × 10^−6^, to stabilize residual variance and convert the inherently multiplicative relationships of gene expression to an additive scale suitable for further statistical analysis. The transformation substantially improved residual normality compared to raw values.

Associations between *FAM153A*, anthropometric variables, and thermogenic adipocyte-associated gene expression were evaluated using Spearman rank correlation (ρ) analysis. Rank correlation coefficients (ρ values) and *p*-values were calculated for each comparison. The Benjamini–Hochberg false discovery rate (FDR) procedure was applied to correct for multiple comparisons across all tests performed.

Partial correlation analyses adjusting for age, sex, and body mass index (BMI) were additionally performed to assess whether observed relationships remained independent of developmental stage and demographic confounders. Partial Spearman correlations were computed using the ordinary least squares (OLS) residual method. Spearman correlation was then applied to the residuals. FDR correction was applied to all partial correlation *p*-values.

To generate an integrated measure of beige adipocyte identity, a composite thermogenic transcriptional score was constructed from the expression levels of four canonical thermogenic adipocyte marker genes (*UCP1*, *TMEM26*, *DIO2*, and *PPARGC1A*). For each gene, the log_10_-transformed expression values were *z*-scored (standardized to mean = 0, SD = 1), and the four *z*-scores were averaged: composite thermogenic transcriptional score = ¼ Σ*z*_i_. A higher composite thermogenic transcriptional score indicates greater thermogenic adipocyte identity. This analysis was restricted to 106 patients with complete measurements across all four genes (complete-case analysis; no missing value imputation was applied). A principal component analysis (PCA)-based sensitivity score was computed on the same log_10_ values; concordance with the z-mean score was assessed by Spearman correlation (ρ = 0.901), confirming method robustness.

Two OLS linear regression models were fitted with log_10_(*FAM153A*) as the outcome:

- Model 1: *FAM153A* ~ composite thermogenic transcriptional score + age + sex + BMI (*n* = 106).
- Model 2: *FAM153A* ~ log_10_(*UCP1*) + log_10_(*TMEM26*) + log_10_(*DIO2*) + log_10_(*PPARGC1A*) + age + sex + BMI (*n* = 106).

Heteroscedasticity-consistent standard errors (HC3) were used in both models. Model fit was assessed by adjusted R^2^ and residual diagnostics. Statistical analyses were performed in Python 3.8 (Python.org) using pandas 2.0.3, NumPy 1.24, SciPy 1.11, scikit-learn 1.3.2, Matplotlib 3.7., and GraphPad Prism (version 5.01). A two-sided *p*-value <0.05 was considered statistically significant throughout all analyses.

## 3. Results

### 3.1. FAM153A Is Associated with Human Preadipocytes and Adipocytes

**Figure 1:**
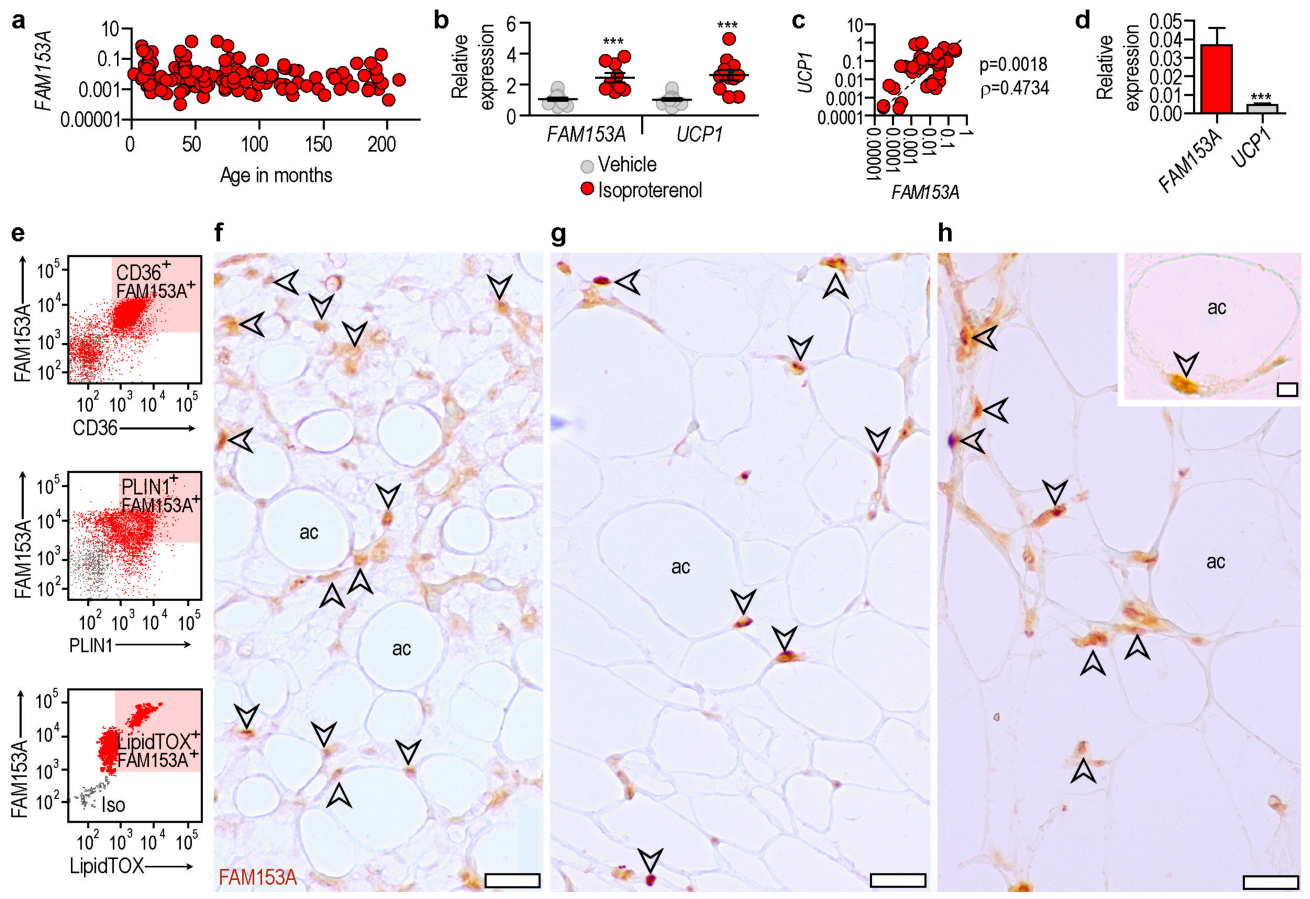
Expression of *FAM153A* mRNA and protein in human adipose tissue. (**a**) Expression of *FAM153A* mRNA in adipose tissue biopsies; each data point represents one patient (*n* = 125). (**b**) Expression of *FAM153A* and *UCP1* in primary adipocytes isolated from adipose tissue biopsies and cultured in vitro. Cells were treated with vehicle or isoproterenol for 4 h, *n* = 10. (**c**) Correlation of *FAM153A* and *UCP1* mRNA expression in primary human adipocytes cultured in vitro, from patients between 6 months and 14 years of age, *n* = 42. Spearman correlation analysis. (**d**) Mean expression levels of *FAM53A* and *UCP1* in adipose tissue biopsies analyzed in this study. (**e**) Fluorescence-assisted flow cytometry analysis of primary human adipocytes isolated from a male patient at 2 years of age. Adipogenic progenitors were labeled with an antibody against CD36, and mature adipocytes with an antibody against perilipin 1 (PLIN1). Lipid droplets were labeled with HCS LipidTOX™ Red (LipidTOX). Iso: isotype control of *FAM153A* labeling. Immunostaining of *FAM153A* in the subcutaneous adipose tissue of a female fetus at the 34th week (**f**), a male infant at 1 month (**g**), and a female child at 12 years of age (**h**). Arrowheads label nuclear *FAM153A* staining, ac: adipocyte, scale bar 30 mm (**f**–**h**) and 5 mm (inlet in panel (**h**)). Representative images. *** *p* < 0.001, Student’s unpaired, 2-tailed *t*-test.

The mRNA level of *FAM153A* was detectable in the adipose tissue in all patients, with a mild, but insignificant decline with increasing age (Figure 1a). Since the expression of genes necessary for adipocyte thermogenesis is triggered by beta adrenergic receptor stimulation, we treated primary human adipocytes with isoproterenol—a ligand for beta adrenergic receptors—in vitro. Transcription of *FAM153A* was increased in response to isoproterenol treatment in a similar magnitude to that of *UCP1*, which encodes the classical thermogenic marker uncoupling protein 1 (Figure 1b). Coherently, the level of *FAM153A* was positively correlated with the level of *UCP1* in primary adipocytes (Figure 1c). While both *FAM153A* and *UCP1* mRNA expression were increased by isoproterenol treatment, *FAM153A* was the more abundant transcript in the bulk adipose tissue (Figure 1d).

Next, we used an antibody raised against human *FAM153A* protein to detect its expression in the adipose tissue. *FAM153A* protein was associated with both preadipocytes and adipocytes, as shown by flow cytometry analysis of the stromal vascular fraction (Figure 1e). *FAM153A* was detectable in both CD36^+^, PLIN1^−^ cells that were lacking lipid droplets, and in PLIN1^+^ and lipid droplet-rich cells (Figure 1e). Consistently with this finding, we detected *FAM153A* immunostaining in adipose tissue sections obtained in the intrauterine life, at birth, and in childhood (Figure 1f–h). *FAM153A* immunostaining was detectable in preadipocyte and adipocyte nuclei (Figure 1h).

### 3.2. FAM153A mRNA Expression Positively Correlates with Thermogenic Gene Expression

**Figure 2:**
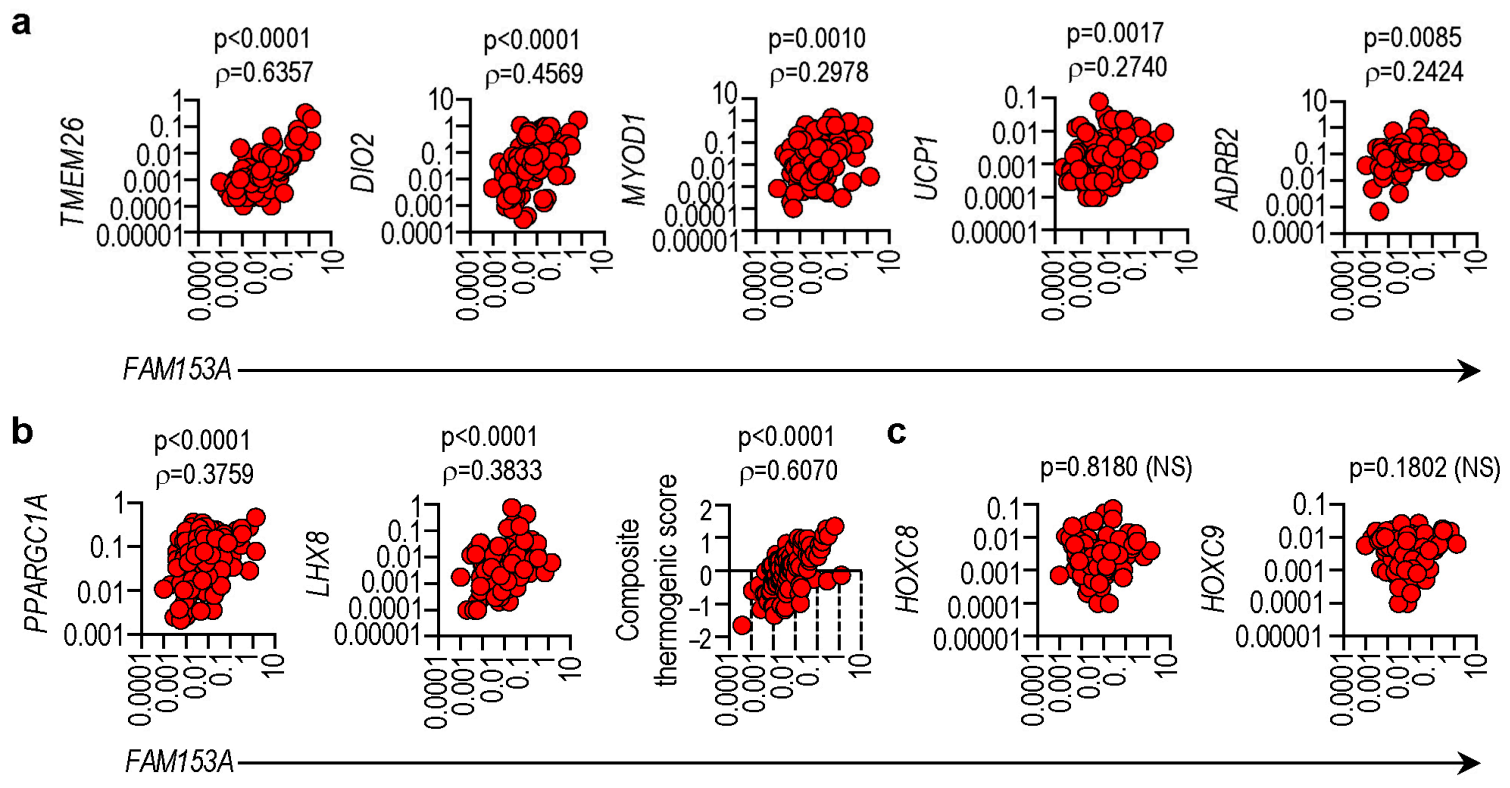
Correlation of *FAM153A* and *UCP1* with the expression of genes associated with thermogenic adipocytes. (**a**) Correlation of *FAM153A* with the expression of *TMEM26*, *DIO2*, *MYOD1*, *UCP1*, and *ADRB2*, *n* = 125. (**b**) Correlation of *FAM153A* with the expression of *PPARGC1A*, *LHX8* and the composite thermogenic score, *n* = 125 and *n* = 106, respectively. (**c**) Correlation of *FAM153A* with the expression of *HOXC8* and *HOXC9*, *n* = 125. NS: non-significant. Spearman correlation analysis.

As a next step, we measured the mRNA level of *FAM153* in adipose tissue biopsies obtained during elective surgeries. The level of *FAM153A* was positively correlated with the expression of genes associated with thermogenesis and mitobiogenesis (Figure 2a,b and Figure S2). These included *UCP1*, *MYOD1* (encoding myogenic differentiation 1, a common signature gene of myoblasts and thermogenic adipocytes), *DIO2* (encoding type II iodothyronine deiodinase), *TMEM26* (encoding transmembrane protein 26), and *PPARGC1A* (encoding peroxisome proliferator-activated receptor gamma coactivator 1-alpha). These genes are actively transcribed in thermogenic adipocytes in both murine and human adipose tissues [22,24,31,32,33]. Moderate positive correlation was found between *FAM153A* and *ADRB2* (encoding beta adrenergic receptor 2), an upstream regulator of thermogenesis in human adipocytes [34]. Moreover, *FAM153A* mRNA expression was positively correlated with the level of *LHX8* mRNA (Figure 2b), encoding Lim Homeobox 8, that is associated with human thermogenic adipocytes [24,35] and has been recently identified as a signal transducer of catecholamine-induced thermogenesis in adipocytes [36].

To further evaluate whether *FAM153A* reflects thermogenic adipocyte identity, a composite thermogenic transcriptional score was generated using established thermogenic adipocyte-associated genes (*UCP1*, *TMEM26*, *DIO2*, and *PPARGC1A*). *FAM153A* expression showed a significant positive correlation with this composite thermogenic score (Spearman ρ = 0.607, *p* < 0.001), confirming that *FAM153A* co-expresses with the thermogenic transcriptional program (Figure 2b). The Spearman correlation with a PCA-based alternative score was ρ = 0.873, confirming the robustness of the equal-weighting *z*-mean approach. *FAM153A* expression differed significantly across tertiles of the composite thermogenic transcriptional score values (Kruskal–Wallis *p* < 0.001), with a monotonic increase from low to high tertile (Figure S4), consistent with *FAM153A* being a marker of thermogenic transcriptional identity.

Coherently, there was a lack of correlation between levels of *FAM153A* and adipocyte maturation markers *HOCX8* and *HOXC9* (encoding homeobox C8 and C9, respectively) [24]. Moreover, there was a lack of correlation between mRNA levels of *FAM153A* and key lipolysis genes *ATGL* (encoding adipose triglyceride lipase) and *MGLL* (encoding monoacylglycerol lipase, also called MAGL) [37,38]. (Figure S3, Table 1).

Adipose tissue functioning is highly dependent on sex and age. Moreover, body mass index (BMI) shows developmental stage specific nadir, peaks, and rebounds in children [39,40,41]. Therefore, partial correlations controlling for age, sex, and BMI were computed via the residual method and multiple testing was corrected using the Benjamini–Hochberg false discovery rate. After adjusting for both sex, age, and BMI as confounding variables, the correlation between *FAM153A* and the expression levels of key thermogenic adipocyte marker genes (*TMEM26*, *DIO2*, *PPARGC1A*, *LHX8*, and *MYOD1*) remained significant (Table 1). These associations were therefore not attributable to demographic confounding. As shown in Table 1, *FAM153A* was a robust indicator of thermogenic gene transcription.

### 3.3. FAM153A mRNA Expression Is Associated with Thermogenic Adipocyte Identity

As a next step, an OLS linear regression model was developed to determine whether the composite thermogenic transcriptional score independently predicted *FAM153A* expression after adjusting for potential confounding factors, including age, sex, and BMI. Importantly, *FAM153A* remained an independent positive predictor of the thermogenic transcriptional program, since the composite thermogenic transcriptional score remained a significant positive predictor of *FAM153A* expression (β = 0.799, 95% CI [0.541, 1.057], *p* < 0.001, adjusted R^2^ = 0.3748) (Table 2, Figure 2b).

When we assessed the potential individual contribution of each thermogenic adipocyte marker to the expression of *FAM153A* in a multivariate linear model, we found that *TMEM26* was the only gene with an individually significant coefficient (β = 0.823, 95% CI [0.445, 1.200], *p* < 0.001, adjusted R^2^ = 0.4817) (Table 3). This indicated that it made a unique contribution to *FAM153A* expression, and that was independent of the expression of other thermogenic adipocyte marker genes. This was consistent with *TMEM26* having the strongest bivariate Spearman correlation (ρ = 0.636) and the strongest partial correlation (ρ = 0.5902) with *FAM153A* expression (Figure 2a). Additionally, *PPARGC1A* was the second relevant independent contributor to *FAM153A* expression (Table 3).

### 3.4. FAM153A Indicates Thermogenic, Small Adipocytes

**Figure 3:**
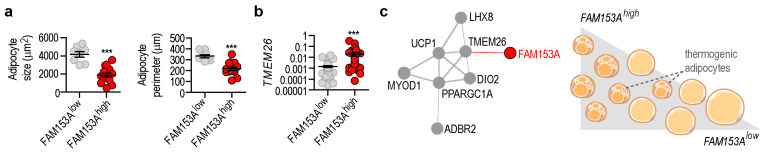
*FAM153A* expression indicates small, thermogenic adipocytes in adipose tissue biopsies. (**a**) Histomorphometry analysis of tissue sections of fat biopsies from patients with low and high *FAM153A* mRNA expression (*FAM153A*^low^, *FAM153A* expression units below 0.0215; *FAM153*^high^, *FAM153A* expression units above 0.0215); each data point represents one patient. (**b**) Expression level of TMEM26 mRNA in fat biopsies from patients with low and high *FAM153A* mRNA expression. *** *p* < 0.001, Student’s unpaired, 2-tailed *t*-test. (**c**) Protein–protein interaction map generated by STRING [42], positioning *FAM153A* associated with the thermogenic gene network. Expression of *FAM153A* in human fat biopsies is proportional with the expression of the thermogenic gene network, and hence serves as a quantitative marker of thermogenic adipocytes.

Samples with *FAM153A* levels above 0.0215 expression units, corresponding approximately to the upper quartile of the cohort, were classified as *FAM153A*^high^, whereas all remaining samples were classified as *FAM153A*^low^. Using this upper-quartile-based descriptive stratification of the samples, we found that adipocyte size was significantly lower in *FAM153A*^high^ adipose tissue samples (Figure 3a). Coherently, *TMEM26* expression was higher in the *FAM153A*^high^ group (Figure 3b). These findings suggest that elevated *FAM153A* expression was associated with a metabolically active adipocyte phenotype and reduced lipid accumulation. In summary, *FAM153A* expression was strongly associated with the thermogenic gene network (Figure 3c), and *FAM153A* mRNA expression was an indicator of presence of thermogenic, small adipocytes in fat biopsies (Figure 3c).

## 4. Discussion

Here, we studied whether the number or activity of thermogenic adipocytes might be estimated by the analysis of a single mRNA species in fat biopsies of children.

Recently, mRNA species have been used a biomarkers and are considered as possible diagnostic tools [43]. Analysis of mRNA offers an earlier detection of gene expression changes than protein analysis. Detection of mRNA is more sensitive and more specific than traditional protein analysis. For instance, in a qPCR-based mRNA analysis there is no need for antigen–antibody interactions and secondary signal amplification—as in immunohistochemistry, flow cytometry, enzyme-linked immunosorbent assay, or Western blotting—reducing non-specific signals and increasing analytical accuracy. Moreover, qPCR allows the analysis of considerably smaller samples than protein-based assays, making it suitable for the analysis of tissue biopsies [44].

Its relatively short turnaround time and compatibility with automated workflows makes qPCR useful in clinical diagnostic procedures, since it allows a rapid diagnosis. It may be easily performed in most clinical laboratories. The qPCR technique has, however, limitations, such as high costs of equipment, susceptibility to inhibition by sample-derived compounds, and the dependence on trained personnel [44].

Today qPCR is used as a diagnostic tool when the diagnostic target is DNA or RNA; for instance, recognition of pathogens, fusion transcripts, determining viral load, or copy-number changes. Therefore, qPCR is used in the diagnosis of infectious diseases (e.g., detecting SARS-CoV-2, influenza A/B or human papilloma virus, or determining HIV RNA viral load [45,46,47,48,49]). It is also suitable for detecting malignancies, such as in the monitoring of minimal residual disease [50]. Moreover, qPCR may be used to detect DNA or RNA biomarkers across clinical samples. The majority of the already-established DNA and RNA biomarkers are related to malignant tumors, such as circulating tumor DNA, circulating free DNA, and microRNA species [51].

Here, we show that *FAM153A* mRNA may be a sensitive indicator of the thermogenic transcriptional program in adipose tissue, hence we suggest that it is an RNA-type biomarker of the thermogenic fat in children. Since mRNA may be purified from tissue biopsies, measuring *FAM153A* mRNA with qPCR may be a useful tool to estimate the thermogenic adipocyte content of adipose tissue biopsies.

Pediatric obesity is associated with an accelerated loss of thermogenic adipocytes [20,52]. Sustained thermogenic activity of the adipose tissue improves insulin sensitivity and may reduce the risk of obesity in children [12]. It is also plausible that the premature loss of thermogenic fat cells may also contribute to the gradual increase of lipid storage and eventually, obesity [21]. Estimating the amount of thermogenic fat hence provides relevant information on adipose tissue health.

Traditionally, UCP1 is considered the golden standard of thermogenic fat, since it is the molecule necessary for mitochondrial thermogenesis, and it constitutes about 10% of the total mitochondrial proteins in thermogenic adipocytes [53]. However, thermogenesis may appear in the lack of UCP1 [54,55], and thermogenic adipocyte identity may be better defined by the co-expression of a set of genes [24,32,35,56]. For instance, co-expression of *TMEM26*, *DIO2*, *MYOD1*, and *PPARGC1A* is associated with thermogenic adipocytes [24,31,32,35,56]. Moreover, a recent study identified two novel gene products in thermogenic adipocytes: potassium voltage-gated channel subfamily C member 2 (*Kcnc2*) and predicted gene 5627 (*Gm5627*), with functions still to be elucidated [57]. Overall, defining thermogenic adipocytes requires the analysis of a gene network, rather than a single gene product. In a diagnostic workflow, however, using a single marker to define thermogenic competence is more feasible, especially when sample size and downstream analysis possibilities are limited.

The association of *FAM153A* with the thermogenic transcriptional identity makes it a suitable candidate for the rapid assessment of thermogenic potential in pediatric adipose tissue biopsies, using bulk tissue samples instead of isolated adipocytes. Measuring *FAM153A* mRNA in fat biopsies would replace the need for measuring the expression of the entire thermogenic gene network, or the flow cytometry analysis of isolated adipocytes.

The biological function of *FAM153A* still remains unknown. It was initially identified as an antigen associated with renal cell carcinoma and termed as renal carcinoma antigen NY-REN-7 [58]. We have previously identified *FAM153A* mRNA expression in adipose tissue of children with early adiposity rebound [15]. *FAM153A* belongs to a family of genes with unknown functions and similar sequences, termed as family with sequence similarity (FAM). Their abnormal expression patterns are associated with various forms of cancer and may have prognostic value [58,59]. For instance, FAM153B, which is a pseudogene, shows compromised expression in gastric cancer and its overexpression inhibits cell proliferation [60]. FAM13A—not to be confused with *FAM153A*—is involved in adipose tissue development. It protects IRS1 from proteasomal degradation and improves adipocyte insulin sensitivity, and the lack of FAM13A exacerbates obesity-related metabolic disorders in mice [61]. Coherently, FAM13A overexpression increases susceptibility of adipocyte precursors to apoptosis and blocks adipogenesis [62]. In turn, knockdown of FAM13A increases adipocyte differentiation from both human and mouse preadipocytes in vitro [63]. These functions of FAM genes may limit adipocyte proliferation and development of mature adipocytes. Although it is too early to speculate on the function of *FAM153A* in adipocytes, the cell cycle inhibiting effects of its cognate proteins suggests that it may limit adipocyte expansion and protect from obesity. Our present study identifies it as an indicator of thermogenic adipose tissue.

## 5. Conclusions

We show that *FAM153A* is a novel biomarker of the human thermogenic transcriptional network. The comprehensive correlation analysis and regression models verified that its expression was positively associated not only with the expression of individual thermogenic genes but also with their coordinated co-expression making it a robust indicator of the thermogenic transcriptional network. It may be measured by qPCR, making it suitable for the rapid assessment of thermogenic competence in fat biopsies.

## Figures and Tables

**Table 1:** Partial Spearman correlations of gene expression values with *FAM153A* mRNA levels, controlled for confounding factors, ranked by partial ρ-values.

Gene Name	Partial ρ-Value	*p*-Value	q-Value	Significance
*TMEM26*	0.590	<0.001	<0.001	True
*DIO2*	0.398	<0.001	<0.001	True
*PPARGC1A*	0.354	<0.001	<0.001	True
*LHX8*	0.354	<0.001	<0.001	True
*MYOD1*	0.245	0.007	0.016	True
*UCP1*	0.190	0.042	0.078	False
*ADRB2*	0.124	0.184	0.254	False
*HOXC9*	0.046	0.621	0.683	False
*HOXC8*	−0.024	0.804	0.804	False
*ATGL*	−0.085	0.369	0.451	False
*MGLL*	−0.171	0.071	0.111	False

q-value refers to false discovery rate adjusted *p*-value.

**Table 2:** Regression coefficients for *FAM153A* expression by composite thermogenic transcriptional score.

Predictor	Beta	SE	*p*	CI Lower	CI Upper
Intercept	−2.225	0.356	<0.001	−2.923	−1.527
Composite thermogenic transcriptional score	0.799	0.132	<0.001	0.541	1.057
Age	−0.018	0.017	0.293	−0.051	0.015
Sex	0.600	0.312	0.057	−0.011	1.211
BMI	0.001	0.021	0.974	−0.0412	0.043

Age, sex, and BMI were considered as confounders. SE: standard error of β, CI: 95% confidence interval (lower and upper bounds).

**Table 3:** Individual contribution model of each thermogenic adipocyte marker gene and anthropometric values to the expression of *FAM153A*.

Predictor	Beta	SE	*p*	CI Lower	CI Upper
Intercept	1.061	0.494	0.034	0.093	2.030
*TMEM26*	0.823	0.193	<0.001	0.445	1.200
*PPARGC1A*	0.204	0.147	0.169	−0.084	0.493
*DIO2*	0.057	0.112	0.615	−0.164	0.277
*UCP1*	0.141	0.138	0.307	−0.128	0.411
Age	−0.012	0.015	0.425	−0.043	0.018
Sex	0.345	0.241	0.155	−0.128	0.818
BMI	−0.009	0.019	0.654	−0.046	0.029

Age, sex, and BMI were considered as confounders. SE: standard error of β, CI: 95% confidence interval (lower and upper bounds).

## Data Availability

The data presented in this study (unprocessed image files, FACS data and qPCR raw data) are available on request from the corresponding author. These data are not publicly available due to privacy restrictions.

## Supplementary Materials

The following supporting information can be downloaded at: https://www.mdpi.com/article/10.3390/cells15151321/s1, Figure S1: Flow cytometry gating strategy; Figure S2: Spearman correlation half-matrix across all measured genes; Figure S3: Correlation of *FAM153A*, ATGL, and MGLL mRNA expression; Figure S4: *FAM153A* levels by the composite thermogenic score tertiles (Kruskal–Wallis test); Table S1: qPCR primer sequences used in the study; Table S2: Characteristics of the qPCR primer pair used for detecting *FAM153A*; Table S3: *FAM153A* transcript variants detected by our assay.

## References

[1] Sørensen T.I.A. (2025). Associations of obesity with co-morbidities in early adult life. Lancet Diabetes Endocrinol..

[2] Lister N.B., Baur L.A., Felix J.F., Hill A.J., Marcus C., Reinehr T., Summerbell C., Wabitsch M. (2023). Child and adolescent obesity. Nat. Rev. Dis. Prim..

[3] The-Lancet-Editorial (2025). Childhood obesity: A global health crisis. Lancet.

[4] Khera A.V., Chaffin M., Wade K.H., Zahid S., Brancale J., Xia R., Distefano M., Senol-Cosar O., Haas M.E., Bick A. (2019). Polygenic Prediction of Weight and Obesity Trajectories from Birth to Adulthood. Cell.

[5] Keller M., Vogel M., Garten A., Svensson S.I.A., Rossi E., Kovacs P., Böttcher Y., Kiess W. (2026). Epigenetics of Childhood Obesity. Horm. Res. Paediatr..

[6] Chami N., Wang Z., Svenstrup V., Obrero V.D., Hemerich D., Huang Y., Dashti H., Manitta E., Preuss M.H., North K.E. (2025). Genetic subtyping of obesity reveals biological insights into the uncoupling of adiposity from its cardiometabolic comorbidities. Nat. Med..

[7] Saba L., Acosta A.J., Kelly A.S., Kumar S. (2026). Obesity Phenotyping in Children and Adolescents: Next Steps Towards Precision Medicine in Pediatric Obesity. Nutrients.

[8] Cartwright B.R., Scherer P.E. (2025). Adipose Tissue as a Target for Precision Medicine Approaches in Childhood Obesity. Diabetes.

[9] Anazco D., Acosta A. (2025). Precision medicine for obesity: Current evidence and insights for personalization of obesity pharmacotherapy. Int. J. Obes..

[10] Alcalá M., Calderon-Dominguez M., Serra D., Herrero L., Viana M. (2019). Mechanisms of Impaired Brown Adipose Tissue Recruitment in Obesity. Front. Physiol..

[11] Gilsanz V., Hu H.H., Kajimura S. (2013). Relevance of brown adipose tissue in infancy and adolescence. Pediatr. Res..

[12] Tews D., Wabitsch M. (2022). Brown Adipose Tissue in Children and Its Metabolic Function. Horm. Res. Paediatr..

[13] Cypess A.M., Kahn C.R. (2010). The role and importance of brown adipose tissue in energy homeostasis. Curr. Opin. Pediatr..

[14] Leitner B.P., Huang S., Brychta R.J., Duckworth C.J., Baskin A.S., McGehee S., Tal I., Dieckmann W., Gupta G., Kolodny G.M. (2017). Mapping of human brown adipose tissue in lean and obese young men. Proc. Natl. Acad. Sci. USA.

[15] Gyurina K., Yarmak M., Sasi-Szabó L., Molnár S., Méhes G., Röszer T. (2023). Loss of Uncoupling Protein 1 Expression in the Subcutaneous Adipose Tissue Predicts Childhood Obesity. Int. J. Mol. Sci..

[16] Sinclair J.C. (1978). Temperature Regulation and Energy Metabolism in the Newborn (Monographs in Neonatalogy).

[17] Gilsanz V., Smith M.L., Goodarzian F., Kim M., Wren T.A., Hu H.H. (2012). Changes in brown adipose tissue in boys and girls during childhood and puberty. J. Pediatr..

[18] Lean M.E., James W.P., Jennings G., Trayhurn P. (1986). Brown adipose tissue uncoupling protein content in human infants, children and adults. Clin. Sci..

[19] Gilsanz V., Chung S.A., Jackson H., Dorey F.J., Hu H.H. (2011). Functional brown adipose tissue is related to muscle volume in children and adolescents. J. Pediatr..

[20] Rockstroh D., Landgraf K., Wagner I.V., Gesing J., Tauscher R., Lakowa N., Kiess W., Bühligen U., Wojan M., Till H. (2015). Direct evidence of brown adipocytes in different fat depots in children. PLoS ONE.

[21] Yu H., Dilbaz S., Coßmann J., Hoang A.C., Diedrich V., Herwig A., Harauma A., Hoshi Y., Moriguchi T., Landgraf K. (2019). Breast milk alkylglycerols sustain beige adipocytes through adipose tissue macrophages. J. Clin. Investig..

[22] Kozak L.P. (2011). The genetics of brown adipocyte induction in white fat depots. Front. Endocrinol..

[23] Ribeiro M.O., Lebrun F.L., Christoffolete M.A., Branco M., Crescenzi A., Carvalho S.D., Negrão N., Bianco A.C. (2000). Evidence of UCP1-independent regulation of norepinephrine-induced thermogenesis in brown fat. Am. J. Physiol. Endocrinol. Metab..

[24] de Jong J.M., Larsson O., Cannon B., Nedergaard J. (2015). A stringent validation of mouse adipose tissue identity markers. Am. J. Physiol. Endocrinol. Metab..

[25] Santosa S., Swain J., Tchkonia T., Kirkland J.L., Jensen M.D. (2015). Inflammatory characteristics of adipose tissue collected by surgical excision vs needle aspiration. Int. J. Obes..

[26] Pincu Y., Yoel U., Haim Y., Makarenkov N., Maixner N., Shaco-Levy R., Bashan N., Dicker D., Rudich A. (2022). Assessing Obesity-Related Adipose Tissue Disease (OrAD) to Improve Precision Medicine for Patients Living with Obesity. Front. Endocrinol..

[27] Hausman D.B., Park H.J., Hausman G.J. (2008). Isolation and culture of preadipocytes from rodent white adipose tissue. Methods Mol. Biol..

[28] Untergasser A., Ruijter J.M., Benes V., van den Hoff M.J.B. (2021). Web-based LinRegPCR: Application for the visualization and analysis of (RT)-qPCR amplification and melting data. BMC Bioinform..

[29] Gao H., Volat F., Sandhow L., Galitzky J., Nguyen T., Esteve D., Åström G., Mejhert N., Ledoux S., Thalamas C. (2017). CD36 Is a Marker of Human Adipocyte Progenitors with Pronounced Adipogenic and Triglyceride Accumulation Potential. Stem Cells.

[30] Blanchette-Mackie E.J., Dwyer N.K., Barber T., Coxey R.A., Takeda T., Rondinone C.M., Theodorakis J.L., Greenberg A.S., Londos C. (1995). Perilipin is located on the surface layer of intracellular lipid droplets in adipocytes. J. Lipid Res..

[31] Timmons J.A., Wennmalm K., Larsson O., Walden T.B., Lassmann T., Petrovic N., Hamilton D.L., Gimeno R.E., Wahlestedt C., Baar K. (2007). Myogenic gene expression signature establishes that brown and white adipocytes originate from distinct cell lineages. Proc. Natl. Acad. Sci. USA.

[32] Sharp L.Z., Shinoda K., Ohno H., Scheel D.W., Tomoda E., Ruiz L., Hu H., Wang L., Pavlova Z., Gilsanz V. (2012). Human BAT possesses molecular signatures that resemble beige/brite cells. PLoS ONE.

[33] Seale P., Kajimura S., Yang W., Chin S., Rohas L.M., Uldry M., Tavernier G., Langin D., Spiegelman B.M. (2007). Transcriptional control of brown fat determination by PRDM16. Cell Metab..

[34] Blondin D.P., Nielsen S., Kuipers E.N., Severinsen M.C., Jensen V.H., Miard S., Jespersen N.Z., Kooijman S., Boon M.R., Fortin M. (2020). Human Brown Adipocyte Thermogenesis Is Driven by β2-AR Stimulation. Cell Metab..

[35] Jespersen N.Z., Larsen T.J., Peijs L., Daugaard S., Homøe P., Loft A., de Jong J., Mathur N., Cannon B., Nedergaard J. (2013). A classical brown adipose tissue mRNA signature partly overlaps with brite in the supraclavicular region of adult humans. Cell Metab..

[36] Gyurina K., Radványi Á., Sasi-Szabó L., Felszeghy E., Rácz E., Méhes G., Kádár A., Fekete C., Röszer T. (2026). Lim Homeobox 8 Is Essential for Beta Adrenergic Stimulation of Thermogenesis in Human Adipocytes. Cells.

[37] Schreiber R., Diwoky C., Schoiswohl G., Feiler U., Wongsiriroj N., Abdellatif M., Kolb D., Hoeks J., Kershaw E.E., Sedej S. (2017). Cold-Induced Thermogenesis Depends on ATGL-Mediated Lipolysis in Cardiac Muscle, but Not Brown Adipose Tissue. Cell Metab..

[38] Jiang M., Huizenga M.C.W., Wirt J.L., Paloczi J., Amedi A., van den Berg R.J.B.H.N., Benz J., Collin L., Deng H., Di X. (2023). A monoacylglycerol lipase inhibitor showing therapeutic efficacy in mice without central side effects or dependence. Nat. Commun..

[39] Wen X., Kleinman K., Gillman M.W., Rifas-Shiman S.L., Taveras E.M. (2012). Childhood body mass index trajectories: Modeling, characterizing, pairwise correlations and socio-demographic predictors of trajectory characteristics. BMC Med. Res. Methodol..

[40] Pomi A.L., Pepe G., Aversa T., Corica D., Valenzise M., Messina M.F., Morabito L.A., Stagi S., Wasniewska M. (2024). Early adiposity rebound: Predictors and outcomes. Ital. J. Pediatr..

[41] Gavin K.M., Bessesen D.H. (2020). Sex Differences in Adipose Tissue Function. Endocrinol. Metab. Clin. N. Am..

[42] Szklarczyk D., Gable A.L., Lyon D., Junge A., Wyder S., Huerta-Cepas J., Simonovic M., Doncheva N.T., Morris J.H., Bork P. (2019). STRING v11: Protein-protein association networks with increased coverage, supporting functional discovery in genome-wide experimental datasets. Nucleic Acids Res..

[43] Sunde R.A. (2010). mRNA transcripts as molecular biomarkers in medicine and nutrition. J. Nutr. Biochem..

[44] Rodrigues T., de Moura B.G., de Miranda G.T.C., Toledo C.L., Bravo L.F., Costa A.D.T. (2026). Application of qPCR testing in clinical diagnostics: A brief review of its history, challenges and perspectives. AIMS Mol. Sci..

[45] Centers for Disease Control and Prevention (CDC) Information on Rapid Molecular Assays, RT-PCR, and Other Molecular Assays for Diagnosis of Influenza Virus Infection. https://www.cdc.gov/flu/hcp/testing-methods/molecular-assays.html.

[46] Linkowska K., Bogiel T., Lamperska K., Marszałek A., Starzyński J., Szylberg Ł., Szwed-Kowalska A., Pawłowska M., Grzybowski T. (2023). Commercially available SARS-CoV-2 RT-qPCR diagnostic tests need obligatory internal validation. Sci. Rep..

[47] Park J., Kwak N., Chae J.-C., Yoon E.-J., Jeong S.H. (2023). A Two-Step Real-Time PCR Method To Identify Mycobacterium tuberculosis Infections and Six Dominant Nontuberculous Mycobacterial Infections from Clinical Specimens. Microbiol. Spectr..

[48] World Health Organization (2021). WHO Guideline for Screening and Treatment of Cervical Pre-Cancer Lesions for Cervical Cancer Prevention: Use of mRNA Tests for Human Papillomavirus (HPV).

[49] Lin B., Han C., Li J.-H., Wang R. (2024). Evaluation of the performance of a qPCR-based assay for HIV-1 viral load determination. PLoS ONE.

[50] Zhang T., Grenier S., Nwachukwu B., Wei C., Lipton J.H., Kamel-Reid S. (2007). Inter-laboratory comparison of chronic myeloid leukemia minimal residual disease monitoring: Summary and recommendations. J. Mol. Diagn. JMD.

[51] Liu L., Mu B.-R., Zhou Y., Wu Q.-L., Li B., Wang D.-M., Lu M.-H. (2026). Research Trends and Development Dynamics of qPCR-based Biomarkers: A Comprehensive Bibliometric Analysis. Mol. Biotechnol..

[52] Ahmed B.A., Varah N., Ong F.J., Blondin D.P., Gunn E., Konyer N.B., Singh N.P., Noseworthy M.D., Haman F., Carpentier A.C. (2022). Impaired Cold-Stimulated Supraclavicular Brown Adipose Tissue Activity in Young Boys with Obesity. Diabetes.

[53] Czech M.P. (2020). Mechanisms of insulin resistance related to white, beige, and brown adipocytes. Mol. Metab..

[54] Kazak L., Chouchani E.T., Jedrychowski M.P., Erickson B.K., Shinoda K., Cohen P., Vetrivelan R., Lu G.Z., Laznik-Bogoslavski D., Hasenfuss S.C. (2015). A Creatine-Driven Substrate Cycle Enhances Energy Expenditure and Thermogenesis in Beige Fat. Cell.

[55] Orci L., Cook W.S., Ravazzola M., Wang M.-y., Park B.-H., Montesano R., Unger R.H. (2004). Rapid transformation of white adipocytes into fat-oxidizing machines. Proc. Natl. Acad. Sci. USA.

[56] Pilkington A.-C., Paz H.A., Wankhade U.D. (2021). Beige Adipose Tissue Identification and Marker Specificity—Overview. Front. Endocrinol..

[57] Onodera K., Hasegawa Y., Yokota N., Tamura S., Kinno H., Takahashi I., Chiba H., Kojima H., Katagiri H., Nata K. (2024). A newly identified compound activating UCP1 inhibits obesity and its related metabolic disorders. Obesity.

[58] Scanlan M.J., Gordan J.D., Williamson B., Stockert E., Bander N.H., Jongeneel V., Gure A.O., Jäger D., Jäger E., Knuth A. (1999). Antigens recognized by autologous antibody in patients with renal-cell carcinoma. Int. J. Cancer.

[59] Deng H., Xu X., Zhang Y., Li Y. (2025). The complex role and molecular mechanism of family with sequence similarity genes in cancer: A comprehensive review. Discov. Oncol..

[60] Yang Y., Ye Y., Liu M., Zheng Y., Wu G., Chen Z., Wang Y., Guo Q., Ji R., Zhou Y. (2022). Family with sequence similarity 153 member B as a potential prognostic biomarker of gastric cancer. Math. Biosci. Eng. MBE.

[61] Wardhana D.A., Ikeda K., Barinda A.J., Nugroho D.B., Qurania K.R., Yagi K., Miyata K., Oike Y., Hirata K.-i., Emoto N. (2018). Family with sequence similarity 13, member A modulates adipocyte insulin signaling and preserves systemic metabolic homeostasis. Proc. Natl. Acad. Sci. USA.

[62] Tang J., Zhou H., Sahay K., Xu W., Yang J., Zhang W., Chen W. (2019). Obesity-associated family with sequence similarity 13, member A (FAM13A) is dispensable for adipose development and insulin sensitivity. Int. J. Obes..

[63] Fathzadeh M., Li J., Rao A., Cook N., Chennamsetty I., Seldin M., Zhou X., Sangwung P., Gloudemans M.J., Keller M. (2020). FAM13A affects body fat distribution and adipocyte function. Nat. Commun..

