# Supplementary material for: FAM153A Is a Novel Biomarker of Human Thermogenic Adipocytes
Source: Cells. 2026 Jul 24;15(15):1321. doi: 10.3390/cells15151321 (PMC13465055; doi:10.3390/cells15151321)
Supplement: Supplementary file 1 [file cells-15-01321-s001.zip › cells-4413449-supplementary.pdf]

Table S1. qPCR primer sequences used in the study (Merck)

FAM153A	fw	CAGACAGTCTCTGAGGAAGCCA
	rev	CTCACCGTTGTAACTGGACAGC
ACTB	fw	CACCSTTGGCAATGAGCGGTTC
	rev	AGGTCTTTGCGGATGTCCACGT
GAPDH	fw	GTCTCCTCTGACTTCAACAGCG
	rev	ACCACCCTGTTGCTGTAGCCAA
LHX8	fw	GGACCAGCTTTACAGCAGATCAG
	rev	CGTCTGCTCAAGCCTGTCCTTT
UCP1	fw	AGTTCCTCACCGCAGGGAAAGA
	rev	GTAGCGAGGTTTGATTCCGTGG
PPARGC1A	fw	CCAAAGGATGCGCTCTCGTTCA
	rev	CGGTGTCTGTAGTGGCTTGACT
MYOD1	fw	CTCCAACTGCTCCGACGGCAT
	rev	ACAGGCAGTCTAGGCTCGACAC
DIO2	fw	TTGAGCCGCTCCAAGTCCACTC
	rev	CTGTACTGGAGACATGCACCAC
TMEM26	fw	GCAGTTTCCACTTGACCTGGCA
	rev	GAAGACGCTGATTCCGATGTTCC
ADRB2	fw	TACCAGAGCCTGCTGACCAAGA
	rev	AGTCACAGCAGGTCTCATTGGC
ATGL	fw	CCCACTTCAACTCCAAGGACGA
	rev	GCAGGTTGTCTGAAATGCCACC
MGLL	fw	GGCATGGTACTCATTTCGCCTC
	rev	GTTTGGCAGCACAAGGTTGAGC
HOXC8	fw	AACTCGTCTCCCAGCCCTCATGT
	rev	TCTAGTTCCAAGGTCTGATACCG
HOXC9	fw	CAGCAAGCACAAAGAGGAGAAGG
	rev	AGTTCCAGCGTCTGGTACTTGG

**Table S2. Characteristics of the qPCR pimer pair used for detecting *FAM153A***	
Forward primer (5'→3')	CAGACAGTCTCTGGAGAAGCCA
Reverse primer (5'→3')	CTCACCGTTGTAACTGGACAGC
Amplicon size	114 bp
Forward primer T_m	61.41 °C
Reverse primer T_m	61.43 °C
Forward primer GC content	54.55%
Reverse primer GC content	54.55%
amplification efficiency (%)	>95

**Table S3.** ***FAM153A*** **transcript variants detected by our assay**	
**Transcript variant**	**RefSeq accession**
X18	XM_047417107.1
X8	XM_017009358.2
X7	XM_017009356.2
X14	XM_017009363.2
X15	XM_017009364.2
X13	XM_017009362.2
4	NM_001394339.1
X18 (alternative assembly)	XM_047417106.1
X17	XM_017009369.2
1	NM_173663.5
X10	XM_017009360.2
X2	XM_017009352.2
X16	XM_017009365.2
X1	XM_017009355.2
X20	XM_006714849.4
X4	XM_017009357.2
X5	XM_017009361.2
X9	XM_017009359.2
X11	XM_011534520.3
5	NM_001413826.1
X19	XM_011534521.4
X6	XM_017009354.2
X3	XM_005265889.4
X24	XM_054352411.1
X23	XM_054352410.1
X4 (new assembly)	XM_054352397.1
X12	XM_054352405.1
X5 (new assembly)	XM_054352398.1
X11 (new assembly)	XM_054352404.1
X3 (new assembly)	XM_054352396.1
X10 (new assembly)	XM_054352403.1
X7 (new assembly)	XM_054352400.1
X9 (new assembly)	XM_054352402.1
X17 (new assembly)	XM_054352413.1
X8 (new assembly)	XM_054352401.1
X6 (new assembly)	XM_054352399.1
X16 (new assembly)	XM_054352409.1
X20 (new assembly)	XM_054352412.1
X15 (new assembly)	XM_054352408.1
X1 (new assembly)	XM_054352394.1
X13 (new assembly)	XM_054352406.1
X14 (new assembly)	XM_054352407.1
X2 (new assembly)	XM_054352395.1
X25	XM_054352412.1

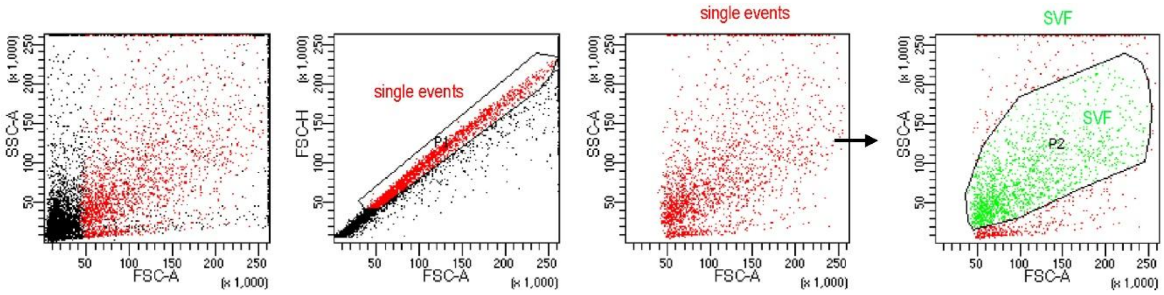

Figure S1. Flow cytometry gating strategy
SSC-A, side scatter area (proportional to cellular internal complexity); FSC-A, forward scatter area (proportional to cell size); FSC-H, forward scatter height (used together with FSC-A for discrimination of cell doublets), SVF: stromal vascular fraction

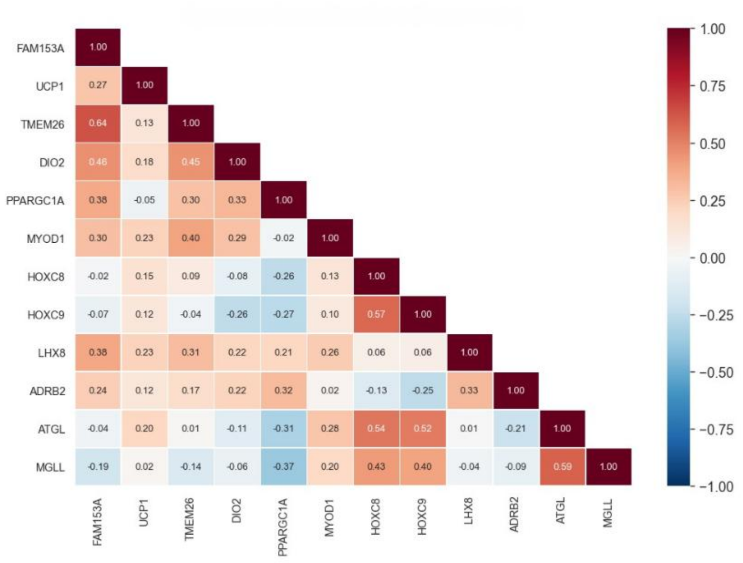

Figure S2. Spearman correlation half-matrix across all measured genes.

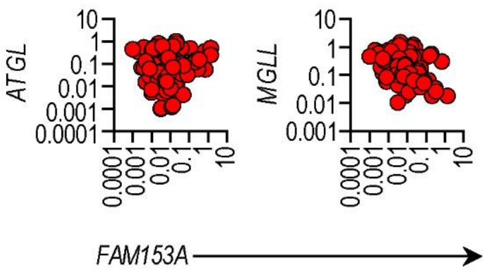

Figure S3. Correlation of *FAM153A*, *ATGL* and *MGLL* mRNA expression

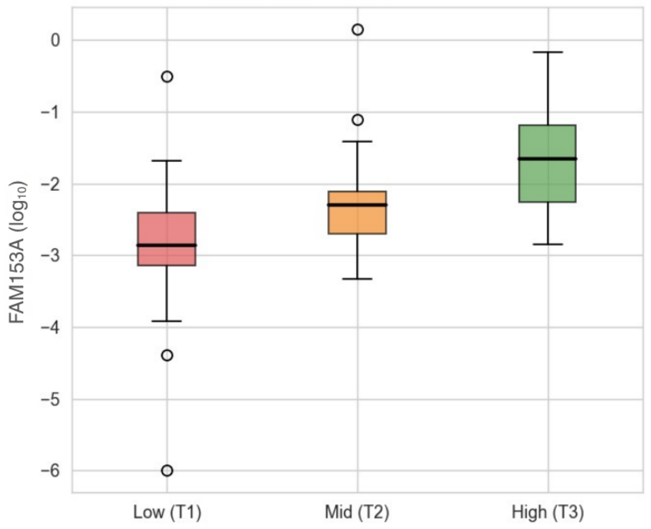

Figure S4. *FAM153A* levels by composite thermogenic score tertiles (Kruskal–Wallis test)
